# Hepatic epithelioid hemangioendothelioma successfully treated with living donor liver transplantation: A case report and literature review

**DOI:** 10.1002/ccr3.2558

**Published:** 2019-12-17

**Authors:** Sotaro Fukuhara, Hiroyuki Tahara, Yoshito Hirata, Kosuke Ono, Michinori Hamaoka, Seiichi Shimizu, Shinji Hashimoto, Shintaro Kuroda, Masahiro Ohira, Kentaro Ide, Tsuyoshi Kobayashi, Hideki Ohdan

**Affiliations:** ^1^ Department of Gastroenterological and Transplant Surgery Graduate School of Biomedical and Health Sciences Hiroshima University Hiroshima Japan

**Keywords:** hepatic epithelioid hemangioendothelioma, liver transplantation, mTOR inhibitor

## Abstract

Hepatic epithelioid hemangioendothelioma is a rare neoplasm with a variable malignant potential and a high risk of recurrence. No general treatment guidelines have been established. Fortunately, we were able to minimize immunosuppressant after liver transplantation because of a full HLA‐matched case. There was no recurrence 1 year after treatment.

## Graphical abstract

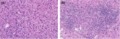

## 1 INTRODUCTION

Epithelioid hemangioendothelioma (EH) is a neoplasm that is derived from vascular endothelial cells and occurs in the lungs, bones, brain, soft tissue, and liver.^1^ Hepatic EH (HEH) is rare, and its malignant potential is variable, with a clinical course between that of a benign hemangioma and that of an angiosarcoma.^1^, ^2^

Hepatic EH often presents with multiple lesions in both hepatic lobes; thus, the most common treatment is liver transplantation (LT). However, no general treatment guidelines have been established because its etiology is unknown. Microvascular or combined macro‐microvascular invasion in the pathological findings has been reported as risk factors of poor prognosis;^3^ however, there is no evidence of an effective adjuvant therapy after LT, with the exception of a few reports. Generally, mammalian target of rapamycin (mTOR) inhibitors are used for suppressing rejection after organ transplantation. They also have an anti‐angiogenetic effect and prevent tumor recurrence. In addition, it is well known that mTOR inhibitors have an antitumor effect, inhibiting an important factor in the mechanism of carcinogenesis and tumor growth.^4^, ^5^

In this case of HEH, the risk of recurrence was considered to be high because tumor vascular invasion was observed in the pathological findings. Herein, we described the case of a patient with HEH treated with living donor liver transplantation and mTOR inhibitors. Notably, this case showed a full HLA match between the donor and recipient. So, we were able to minimize immunosuppressant after liver transplantation, suggesting that is convenient for the suppression of tumor recurrence.

## 2 CASE PRESENTATION

**Figure 1:**
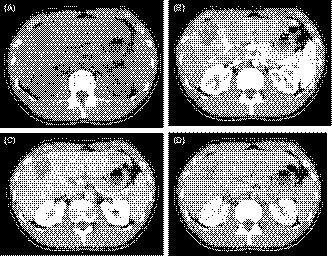
A, Plain computed tomography (CT) revealed multiple tumors with low‐density areas in both hepatic lobes. B‐D, Contrast‐enhanced CT showed the tumor with a slight circular enhancement in the early phase. The enhancement was prolonged to the delayed phase, up to 40 × 46 mm in size

**Figure 2:**
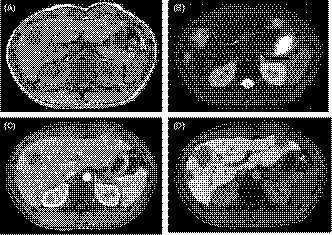
A and B, Magnetic resonance imaging (MRI) showed the tumor with a hypointensity on the T1‐weighted images and a hyperintensity on the T2‐weighted images. C and D, The dynamic MRI study showed the tumor with a heterogeneous enhancement in the early phase and a defect of enhancement in the Kupffer phase

**Figure 3:**
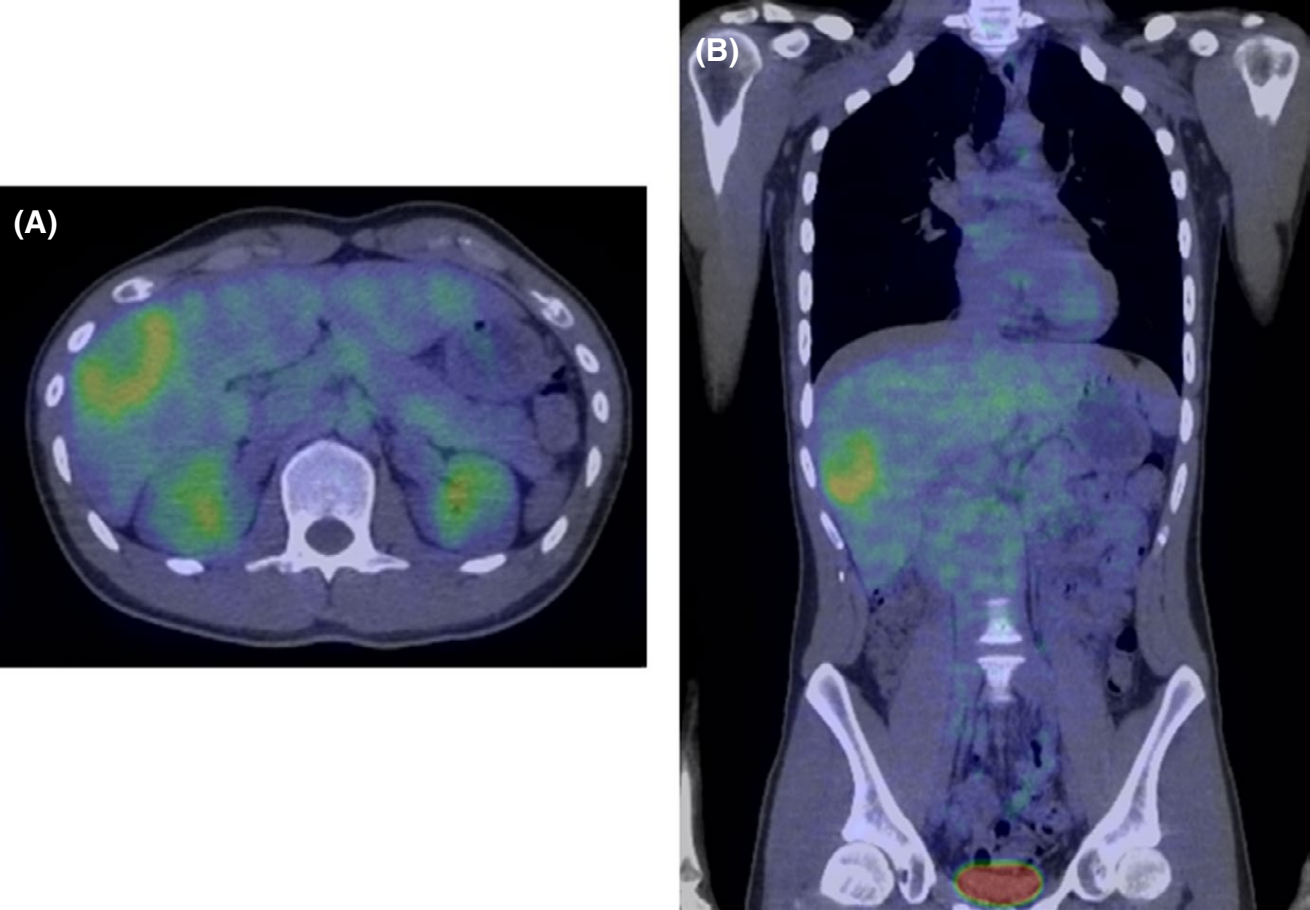
A and B, Fluorin‐18 fluorodeoxyglucose positron emission tomography CT revealed that the tumors had a high accumulation, with a maximum standardized uptake value of 4.9, unlike the other organs

**Figure 4:**
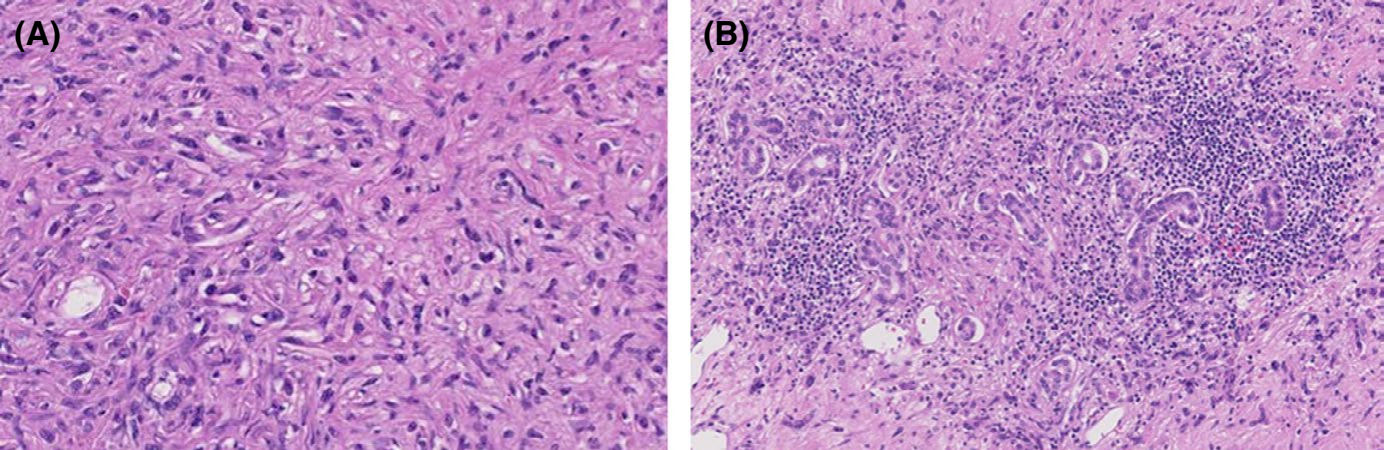
A and B, The histopathological findings revealed that the epithelioid cells were infiltrating the hepatic sinusoids invasively or substitutability. The tumor cells also infiltrated the portal vein and hepatic vein

**Figure 5:**
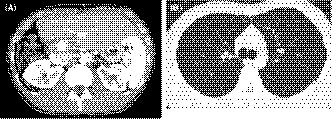
A and B, Contrast‐enhanced CT conducted 12 mo after liver transplantation showed no finding of recurrence or metastasis in the liver graft or the other organs

A 25‐year‐old man who complained of general fatigue was referred to our hospital. He had no past history of serious illness, surgery, or hospitalization. Computed tomography (CT) revealed multiple low‐density areas with a slight circular enhancement in both hepatic lobes, up to 40 × 46 mm in size (Figure 1). Magnetic resonance imaging (MRI) showed the multiple hepatic nodules with hypointensity on the T1‐weighted images and mild hyperintensity on the T2‐weighted images, and a heterogeneous enhancement on the dynamic study (Figure 2). Fluorine‐18 fluorodeoxyglucose positron emission tomography CT (FDG‐PET/CT) revealed a mild‐to‐moderate FDG uptake in the multiple hepatic nodules, with a maximum standardized uptake value (SUVmax) of 4.9 (Figure 3). Tumor markers, including α‐fetoprotein, protein induced by vitamin K absence or antagonist‐II, carcinoembryonic antigen, and carbohydrate antigen 19‐9, were within normal ranges. The possibility of a malignant hepatic tumor, including malignant lymphoma, intrahepatic cholangiocarcinoma, sarcoma, and other tumors with malignant potential, could not be completely excluded due to the increased FDP uptake on FDG‐PET/CT; therefore, we performed a laparoscopic partial liver resection for definitive diagnosis. The histopathological findings revealed that the epithelioid cells were infiltrating the hepatic sinusoids invasively or substitutability (Figure 4A). Immunohistochemically, the tumor cells were positive for CD31, CD34, and factor XIII. Based on these findings (Figure 4C,D), the multiple hepatic tumors were diagnosed as hepatic epithelioid hemangioendothelioma. There was no evidence of extrahepatic lesions in the imaging and operative findings, and the multiple tumors were located in both hepatic lobes, suggesting they were unresectable. Several reports had recommended liver transplantation as a radical treatment in cases of HEH without extrahepatic tumors. For these reasons, living donor liver transplantation was performed with the approval of the Institutional s Committee. A left lobe graft from the patient's brother was used for the living donor liver transplantation, with an identical HLA and blood type. The intraoperative peritoneal lavage cytology was negative. Histopathologically, there was no lymph node or extrahepatic metastasis; however, tumor invasion to the portal vein and hepatic vein was observed (Figure 4B). Immunosuppression was maintained using tacrolimus and everolimus. We selected everolimus in combination with reduced tacrolimus therapy because of the antitumor effect of everolimus. In addition, because of the complete donor‐recipient HLA match (Class I [A, B, C] and Class II [DRB1, DQB1] haplotypes), the immunosuppressant dose could be reduced more than usual. The postoperative course in the recipient was uneventful, and he was discharged on the thirtieth day after the liver transplantation, without evidence of rejection. At the 12‐month follow‐up, there was no recurrence or metastases on the CT scan (Figure 5).

## 3 DISCUSSION

Epithelioid hemangioendothelioma is a neoplasm derived from vascular endothelial cells. It was first described in 1982.^1^ It may occur in the lungs, bones, brain, soft tissue, and the liver, and its malignant potential is variable, with a clinical course between that of a benign hemangioma and that of an angiosarcoma.^1^, ^2^ Primary HEH is a rare neoplasm with an incidence of one per million population.^6^ No general treatment guidelines have been established for HEH due to its unknown etiology. The common treatment includes LT, liver resection (LR), chemotherapy, and radiotherapy. There are several reports comparing the outcomes for each treatment, and we summarized it (Table 1).^2^, ^7^

**Table 1:** Summary of treatment outcomes of hepatic epithelioid hemangioendothelioma

Author	Year	Overall survival					
			LT%	LR%	No treatment%	Chemotherapy or radiotherapy%	Chemotherapy or no treatment%
Mehrabi et al	2006	1‐y 5‐y	96 54.5	100 75	39.3 4.5	73.3 30	
Grotz et al	2010	1‐y 5‐y	91 73	100 86			57 29

Abbreviations: LR, liver resection; LT, liver transplantation.

Mehrabi et al reviewed 434 cases of HEH in 2006. They described that the most common treatment method was LT (44.8%), followed by no treatment (24.8%), chemotherapy or radiotherapy (21%), and LR (9.4%). The respective 1‐year and 5‐year survival rates were 96% and 54.5% for LT, 39.3% and 4.5% for no treatment, 73.3% and 30% for chemotherapy or radiotherapy, and 100% and 75% for LR.^2^ Lerut et al reported 59 HEH cases in 2007, with a 5‐year post‐LT overall survival rate of 83% and a disease‐free survival rate of 82%. The recurrence rate was 24% at an average of almost 50 months after LT. Involvement of both hepatic lobes was seen in 86% of the patients. In addition, the authors found that a microvascular or combined macro‐microvascular invasion significantly affected the survival after LT, but the existence of extrahepatic diseases did not. The 5‐year survival rate in the cases of HEH with extrahepatic diseases was 80%.^3^ Therefore, LR is possible when the HEH is a localized lesion and not possible in the case of multiple HEH lesions in both hepatic lobes. Because the survival rate after LT is better comparatively and HEH is often regarded as an unresectable condition, liver transplantation could be considered as one of the best treatments. In addition, there are studies wherein LT was adopted as a first‐line treatment even when extrahepatic diseases were present.^8^, ^9^ However, in some cases of HEH with extrahepatic diseases, recurrence, metastasis, or death occurred in a short period, within 1 year after LT.^10^, ^11^, ^12^ Further investigation on the significance of LT for HEH with extrahepatic diseases is clearly necessary. There is also not any evidence of an effective adjuvant therapy after LT, with the exception of several case reports, as follows.

In some of these reports, a rapid course to death within months of onset is described, while in others, a long‐term survival without any treatment (maximum period of 28 years) is reported.^13^, ^14^, ^15^ The etiology of HEH is unknown, and the evaluation of its malignant potential is also difficult. Microvascular or combined macro‐microvascular invasion have been reported as prognostic factors, and the pre‐LT waiting time (120 days or less) and hilar lymph node invasion were reported as risk factors for recurrence after LT.^16^ Additional treatment after LT may be necessary for advanced HEH, such as the cases with vascular invasion. Successful treatment with chemotherapy has been reported, using sorafenib^17^, ^18^ and thalidomide,^19^ doxorubicin,^20^ cyclophosphamide,^21^ and interferon.^22^ However, all reports have a small number of cases, and the effect as an established adjuvant therapy after surgery is uncertain.

We summarized 14 studies that reported on LT for HEH (Table 2).^8^, ^9^, ^10^, ^11^, ^12^, ^22^, ^23^, ^24^, ^25^, ^26^, ^27^, ^28^, ^29^, ^30^ While there were cases where death occurred shortly after LT due to early recurrence, there were also cases of long‐term survival even if recurrence was observed after LT. In the cases with long‐term survival, the time to recurrence was longer; thus, the tumor's malignant potential may have been lower. In contrast, in the cases where the time to recurrence was shorter, within a few months, the additional treatment after the recurrence, such as chemotherapy, was ineffective regardless of the existence of extrahepatic diseases at the time of diagnosis, and most of those patients died early. It seems that there was a high risk of recurrence in these cases, although it was not clear, because a vascular infiltration was not described in most. Therefore, it may be better to perform adjuvant therapy after LT in such aggressive cases, before disease recurrence. In our case, tumor vascular invasion was observed; thus, the risk of recurrence was considered to be high. Accordingly, we planned to devise a regimen of immunosuppressant therapy after LT. To the best of our knowledge, there has been no report on the use of mTOR inhibitors as an immunosuppressant therapy after LT for HEH. It is well known that mTOR inhibitors have antitumor effects, inhibiting an important factor in the mechanism of carcinogenesis and tumor growth.^4^, ^5^ Stacchiotti et al reported a retrospective case‐series analysis of 18 patients with an advanced EH treated with the mTOR inhibitor sirolimus. They did not include LT cases. The tumor had spread to multiple organs, and the original site could not be identified. A clinical benefit was achieved in 56% of the patients, and the antitumor effect of sirolimus was reported to have continued for more than 2 years in four patients. In addition, they concluded that the pleural effusion deterioration was associated with the disease progression and that sirolimus may not be effective in more aggressive cases, as observed in those with a worsening pleural effusion, but it may stabilize the EH in advanced cases.^31^ Generally, mTOR inhibitors are used for suppressing rejection after LT. They have an anti‐angiogenetic effect and prevent tumor recurrence. Previous studies have described that mTOR inhibitors are effective in preventing the recurrence and improving the survival rate after LT for hepatocellular carcinoma.^32^, ^33^, ^34^ In our case, considering that the risk of recurrence was high due to the tumor vascular invasion, we used the mTOR inhibitor everolimus in combination with a reduced dose of tacrolimus to aim at not only immunosuppression, but also an antitumor effect after LT. Fortunately, we were able to minimize these immunosuppressants after liver transplantation because of a full HLA‐matched case. Although there was no recurrence or distant metastasis at the 12‐month follow‐up after LT, a long‐term observation is necessary in the future.

**Table 2:** Summary of 14 studies of hepatic epithelioid hemangioendothelioma treated with liver transplantation

Author/Year	Age/Sex	Vascular invasion	Follow‐up period (months)	Time to recurrence (months)/Location	Time to death (months)	Steroid	CNI	EVR	Extrahepatic disease	Treatments
Yalin Tan, et al/2018	30/M	Observed	12	3/Bone	12	NA	NA	NA	Spleen	LT/splenectomy
Antonio, et al/2015	NA/F	NA	84	None	survival	Used	Used	Not used	None	LT
Rudo, et al/2014	64/F	NA	12	12/Liver, spleen	12	Not used	Used	Not used	None	LT/thalidomide
Hasegawa, et al/2006	36/F	NA	8	3/Liver, bone	8	Used	Used	Not used	Spleen	LT/splenectomy/tegafur‐uracil
Lerut, et al/2004	26/M	NA	56	None	survival	Not used	Used	Not used	Brain	Radiotherapy/TACE/resection of brain/LT
	26/M	NA	41	None	survival	Not used	Used	Not used	Hilar LN	LT
	25/F	NA	166	156/Breast	NA	Not used	Used	Not used	None	LT/resection of breast
	42/M	NA	165	None	survival	Not used	Used	Not used	None	LT
	45/F	NA	99	None	survival	Not used	Used	Not used	None	LT
	24/F	NA	22	None	survival	Not used	Used	Not used	None	LT
Simpson, et al/2003	36/F	NA	36	None	survival	NA	NA	NA	None	LT
Peter, et al/2003	46/M	NA	36	None	survival	NA	NA	NA	None	Chemoembolization/LT
Kayer, et al/2002	21/F	NA	2	2/Intrapelvic	16	Used	Used	Not used	None	LT/interferon/5‐FU
Ben‐Haim, et al/1999	40/M	NA	9	9/Liver	9	Used	Used	Not used	None	LT
	25/F	NA	96	48/Liver	NA	Used	Used	Not used	Diaphragm, peritoneum	LT/5‐FU
	53/M	NA	12	None	survival	Used	Used	Not used	None	LT
	34/M	NA	32	None	survival	Used	Used	Not used	Spleen, extrahepatic LN	LT
	61/M	NA	NA	6/Liver, Bone	6	Used	Used	Not used	None	LT
Hung, et al/1998	27/M	NA	24	None	survival	NA	NA	NA	None	LT
Demetris, et al/1998	46/F	NA	41	36/Liver	NA	NA	NA	NA	None	LT
Stadt, et al/1989	34/M	NA	20	20/Liver	NA	Used	Used	Not used	None	LT
Keller, et al/1989	27/F	Observed	134	None	survival	NA	NA	NA	Omentum, lungs	LT
	29/F	None	30	None	survival	NA	NA	NA	Hilar LN	LT
	29/M	None	29	None	survival	NA	NA	NA	Hilar LN	LT
	24/F	None	29	None	survival	NA	NA	NA	Hilar LN	LT
	40/F	None	12	None	survival	NA	NA	NA	None	LT
	33/F	Observed	65	56/Mediastinum	NA	NA	NA	NA	None	LT
	26/F	Observed	16	12/Lungs	16	NA	NA	NA	None	LT/chemoradiotherapy
	30/M	Observed	31	20/Mediastinum	NA	NA	NA	NA	Hilar LN	LT
	37/M	Observed	27	17/Lungs	NA	NA	NA	NA	Hilar LN	LT
	54/M	None	5	3/Bone	NA	NA	NA	NA	Lungs	LT
Marino, et al/1988	NA	NA	132	None	survival	NA	NA	NA	Lungs, diaphragm, pleura	LT/resection of lungs
	NA	NA	48	None	survival	NA	NA	NA	None	LT
	NA	NA	16	12/Liver, lungs	16	NA	NA	NA	None	LT
	NA	NA	24	18/Lungs, Mediastinum	NA	NA	NA	NA	Hilar LN, bile duct	LT
	NA	NA	16	None	survival	NA	NA	NA	Lungs, extrahepatic LN	LT/resection of lungs
	NA	NA	16	None	survival	NA	NA	NA	Hilar LN	LT
	NA	NA	15	None	survival	NA	NA	NA	Extrahepatic LN	LT
	NA	NA	9	None	survival	NA	NA	NA	None	LT
	NA	NA	12	None	survival	NA	NA	NA	None	LT
	NA	NA	3	2/Liver, lungs	3	NA	NA	NA	None	LT

Abbreviations: CNI, calcineurin inhibitor; EVR, everolimus; LN, lymph node; LT, liver transplantation; NA, not available; TACE, transcatheter arterial chemoembolization.

## 4 CONCLUSION

We described a case of multiple HEH in both hepatic lobes treated with living donor transplantation. Considering that the risk of recurrence was high due to the tumor vascular invasion, we used everolimus in combination with a reduced dose of tacrolimus after LT to achieve an antitumor effect. Also, we were able to manage these immunosuppressants to a minimum because of a full HLA‐matched case. This may be useful in suppressing the tumor recurrence. The patient has maintained a good condition a year after LT; however, further careful observation is required in the future.

## CONFLICT OF INTEREST

None of the authors has any conflicts of interest to disclose.

## AUTHOR CONTRIBUTIONS

SF: reviewed the literature, wrote the manuscript, and analyzed the data. HT: reviewed the literature, wrote the manuscript, and interpreted the data. YH: acquired the data and involved in the operation. KO: acquired the data and involved in the operation. MH: acquired the data and involved in the operation. SS: acquired the data and involved in the operation. SH: acquired the data and involved in the operation. SK: acquired the data and involved in the operation. MH: acquired the data and involved in the operation. KI: acquired the data and involved in the operation. TK: acquired the data and involved in the operation. HO: contributed to the review of literature, improved the manuscript, and involved in the operation.

## ETHICAL APPROVAL

All procedures used in this research were approved by the al Committee of Hiroshima University Hospital. Written informed consent was obtained from the patient for the publication of this case report and any accompanying images. A copy of the written consent form is available for review at request.

## GUARANTOR

Hiroyuki Tahara has accepted full responsibility for this work and the decision to publish it.
